# *Helichrysum populifolium* Compounds Inhibit MtrCDE Efflux Pump Transport Protein for the Potential Management of Gonorrhoea Infection

**DOI:** 10.3390/ijms252413310

**Published:** 2024-12-11

**Authors:** Vhangani E. Mulaudzi, Idowu J. Adeosun, Adeniyi T. Adewumi, Mahmoud E. S. Soliman, Sekelwa Cosa

**Affiliations:** 1 Department of Biochemistry, Genetics and Microbiology, University of Pretoria, Private Bag X20, Hatfield, Pretoria 0028, South Africa; u19157623@tuks.co.za (V.E.M.); u21747050@tuks.co.za (I.J.A.); 2 Molecular Bio-Computation and Drug Design Laboratory, School of Health Sciences, University of KwaZulu-Natal, Durban 4000, South Africa; jesutomisin0707@gmail.com (A.T.A.); soliman@ukzn.ac.za (M.E.S.S.)

**Keywords:** efflux pumps, *Helichrysum populifolum*, molecular modeling, multi-drug resistance, *Neisseria gonorrhoeae*, phytochemicals

## Abstract

The progressive development of resistance in *Neisseria gonorrhoeae* to almost all available antibiotics has made it crucial to develop novel approaches to tackling multi-drug resistance (MDR). One of the primary causes of antibiotic resistance is the over-expression of the MtrCDE efflux pump protein, making this protein a vital target for fighting against antimicrobial resistance (AMR) in *N. gonorrhoeae*. This study was aimed at evaluating the potential MtrCDE efflux pump inhibitors (EPIs) and their stability in treating gonorrhoea infection. This is significant because finding novel EPIs would allow for the longer maintenance of antibiotics at therapeutic levels, thereby prolonging the susceptibility of currently available antibiotics. A virtual screening of the selected *Helichrysum populifolium* compounds (4,5-dicaffeoylquinic acid, apigeninin-7-glucoside, and carvacrol) was conducted to evaluate their potential EPI activity. An integrated computational framework consisting of molecular docking (MD), molecular mechanics generalized born, and surface area solvation (MMGBSA) analysis, molecular dynamics simulations (MDS), and absorption, distribution, metabolism, and excretion (ADME) properties calculations were conducted. Of the tested compounds, 4,5-dicaffeoylquinic acid revealed the highest molecular docking binding energies (−8.8 kcal/mol), equivalent MMGBSA binding free energy (−54.82 kcal/mol), indicative of consistent binding affinity with the MtrD protein, reduced deviations and flexibility (root mean square deviation (RMSD) of 5.65 Å) and, given by root mean square fluctuation (RMSF) of 1.877 Å. Carvacrol revealed a docking score of −6.0 kcal/mol and a MMGBSA computed BFE of −16.69 kcal/mol, demonstrating the lowest binding affinity to the MtrD efflux pump compared to the remaining test compounds. However, the average RMSD (4.45 Å) and RMSF (1.638 Å) of carvacrol-bound MtrD protein showed no significant difference from the unbound MtrD protein, except for the reference compounds, implying consistent MtrD conformation throughout simulations and indicates a desirable feature during drug design. Additionally, carvacrol obeyed the Lipinski rule of five which confirmed the compound’s drug-likeness properties making it the most promising EPI candidate based on its combined attributes of a reasonable binding affinity, sustained stability during MDS, its obedience to the Lipinski rule of five and compliance with drug-likeness criteria. An in vitro validation of the potential EPI activities of *H. populifolium* compounds confirmed that 4,5-dicaffeoylquinic acid reduced the expulsion of the *bis*-benzimide dye by MtrCDE pump, while carvacrol showed low accumulation compared to other compounds. While 4,5-dicaffeoylquinic acid demonstrated the highest binding affinity in computational analysis and an EPI activity in vitro, it showed lower stability compared to the other compounds, as indicated in MDS. This leaves carvacrol, as a better EPI candidate for the management of gonorrhoea infection.

## 1. Introduction

Multi-drug resistance (MDR) in *Neisseria gonorrhoea* strains has currently developed into a serious health concern, especially looking at the treatment of gonorrhoea infection [1]. The World Health Organization (WHO) approximates over 87 million gonorrhoea infections worldwide annually [2], while South Africa (SA) reports about 2 million cases per annum, contributing about 2% of the worldwide burden [3]. Treatment of this infection poses a challenge due to the development of resistance to the available antibiotics [4]. What is more concerning is that some *N. gonorrhoeae* strains have been reported to be resistant to the currently used dual therapy treatment, which comprises azithromycin and ceftriaxone. Resistance to these antibiotics has been reported to appear in either one or both antibiotics, resulting in ineffective treatment [2].

In response, several novel antibiotics for gonorrhoea infection treatment are under investigation. Gepotidacin, an oral antibiotic in Phase 3 clinical trials, has demonstrated promising efficacy against uncomplicated urogenital gonorrhea through a unique mechanism of action that targets DNA replication enzymes [4]. Similarly, zoliflodacin, another antibiotic in advanced clinical trials, operates via a distinct mechanism targeting bacterial DNA synthesis and has shown effectiveness against resistant strains [5]. Other antibiotics being explored include solithromycin, a next-generation macrolide with enhanced activity against resistant gonococcal strains, and delafloxacin, a fluoroquinolone with potential applications for resistant infections [6]. These developments demonstrate the urgent need for novel therapeutic options to address the rising threat of multi-drug-resistant *N. gonorrhoeae*.

Analyzing the mechanisms that the pathogen utilizes to elicit antibiotic resistance brings forth new targets in drug discovery [1]. The antimicrobial mechanisms used by *N. gonorrhoeae* include antibiotic inactivation, reduction of antimicrobial concentration, drug target structure modifications, decreased cell-membrane permeability, and extrusion of antibiotics via various efflux pumps (EPs) [2].

The efflux pumps (EPs) are cytoplasmic membrane-embedded proteins that regulate what goes into the cell from the external environment [7]. They can be classified into five classes, which include the ATP binding cassette (ABC), major facilitator proteins, small multi-drug efflux proteins, multi-drug and toxic compound extrusion (MATE), and resistant nodulation (RND) families [1,7]. *N. gonorrhoeae* is known to harbor four EPs: MacAB, NorM, FarAB, and MtrCDE [8]. The multiple transferable resistant (MtrCDE) protein forms part of the RND family. It is one of the well-studied and characterized efflux pumps in *N. gonorrhoeae*, while the remaining three are still under intense research investigations [8]. The MtrCDE is a tripartite system that consists of an outer membrane (OM) channel protein MtrE, the membrane fusion protein MtrC, and an inner membrane (IM) protein, MtrD [7,8]. The MtrD protein of *N. gonorrhoeae* is a homolog of the AcrB protein of *Escherichia coli*, MexB protein of *Pseudomonas aeruginosa*, and MexB protein of *Acetinobacter baumanii*, which interact with antibiotics in the inner membrane [8]. Targeting the MtrD part of the MtrCDE protein proves to be essential, as it binds and extrudes antimicrobials, including antibiotics used to treat gonorrhoea infection, substantiating its high degree of clinical relevance [9].

Furthermore, the MtrCDE protein has a broad-spectrum efflux of antimicrobial agents (AAs), including several antibiotics, dyes, organic salts, and fatty acids out of the cell [8]. It also plays a vital role in the modulation of the host defenses, permitting the pathogen to survive in the unfavorable host-cell environment [10]. Further reports suggest that the MtrCDE protein is involved in cellular activities, such as cellular adherence and colonization, which play a role during the initiation of pathogenesis [1]. This makes MtrCDE an excellent therapeutic target in developing EPIs to combat gonorrhoea infection.

The use of phytochemicals proves to be a promising source of EPIs due to their structural composition, such as alkaloids, flavonoids, terpenoids, and phenolic compounds [11]. Their active inhibitory potential, such as antimicrobial, anti-inflammatory, antioxidant, EPI activity, and others, are attributed to the medicinal plants growing in diverse environments with high microbial populations, thereby enhancing their synthesis for the plant’s protection [11]. Additionally, in comparison to synthetic compounds, phytochemical compounds exhibit fewer side effects when compared to synthetic compounds [12], making them attractive candidates for the development of EPIs. For this reason, *Helichrysum populifolium* compounds were evaluated.

The evaluated phytochemical compounds included 4,5-dicaffeoylquinic acid, apigenin-7-glucoside, and carvacrol from *Helichrysum* genus which were selected based on their reported anti-virulence and potential EPI activity [13,14,15]. The presence of 4,5-dicaffeoylquinic acid was confirmed after conducting liquid chromatography ion trap time of flight mass spectrometry (LC-IT-TOF-MS) by Heyman et al. [13], while apigenin-7-glucoside, and carvacrol were confirmed to be present in *H. populifolium* by the studies conducted by [14,15].

A computer-based screening approach was conducted to evaluate these compounds for their potential EPI activity. This is a significant approach to the search for antimicrobial drugs because it allows for the rapid selection of the most relevant target ligands from a library of molecules [16]. This results in both speeding up the drug development processes and narrowing the number of potential ligands to be screened for efflux pump inhibitory (EPI) activity during further studies [17]. Additionally, computer-based methods can uncover structural motifs in compounds that have not been previously explored, leading to the exploration of new chemical space and the development of novel classes of antimicrobial agents, potentially overcoming resistance mechanisms [18]. Hence, this study was aimed at evaluating potential MtrCDE protein inhibitors from secondary metabolites derived from the *Helichrysum* genus, using a computer-based approach, to treat gonorrhoae infection and reduce antibiotic resistance.

## 2. Results

### 2.1. Crystal Structure of the MtrCDE Efflux Pump Protein

**Figure 1:**
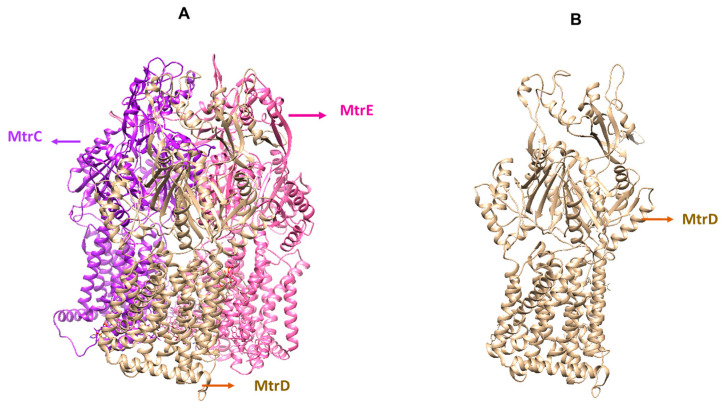
(**A**) Crystal structure of MtrCDE efflux pump protein from *Neisseria gonorrhoea* with its distinguished chains. (**B**) The crystal structure of the MtrD chain of the MtrCDE efflux pump used in this study for molecular docking and molecular dynamic simulations assays, which consists of the proximal and distal multi-drug-binding sites.

The crystal structure of the MtrCDE efflux pump (see Figure 1) was obtained from Protein Data Bank. Molecular docking and simulations were conducted on the MtrD chain of the MtrCDE efflux pump. The MtrD chain of the pump was used because it contains multi-drug-binding sites that allow the extrusion of drugs before they reach their target (see Figure 1).

### 2.2. Molecular Docking of the Studied Compounds

The potential binding and affinity of *H. populifolium* compounds against the crystallized structure of the MtrD protein (resolution 3.02 Å)(PDB ID: 6VKS) in *N. gonorrhoea* obtained from Protein Data Bank (PDB) was evaluated [7]. A cut-off MD score between −5 and −15 kcal/mol was set up to categorize compounds with good docking scores and potential EPI activity. Phenylalanine-arginine-β-naphylamide (PaβN) and quercetin were used as reference compounds as they are well-known EPIs [19,20]. Based on the findings, 4,5-dicaffeoylquinic acid, apigenin-7-glucoside, and carvacrol revealed docking scores of −8.8 kcal/mol, −8.6 kcal/mol, and −6.0 kcal/mol, respectively, comparable to the scores obtained by PaβN, and quercetin which were −8.2 kcal/mol and −8.1 kcal/mol (Table 1), respectively.

**Figure 2:**
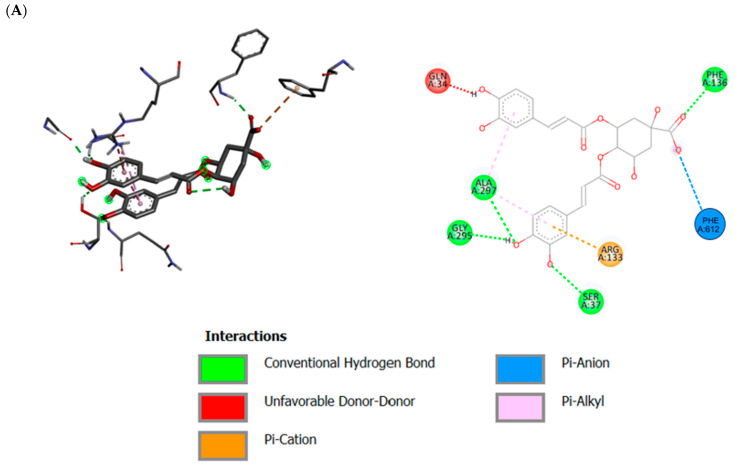

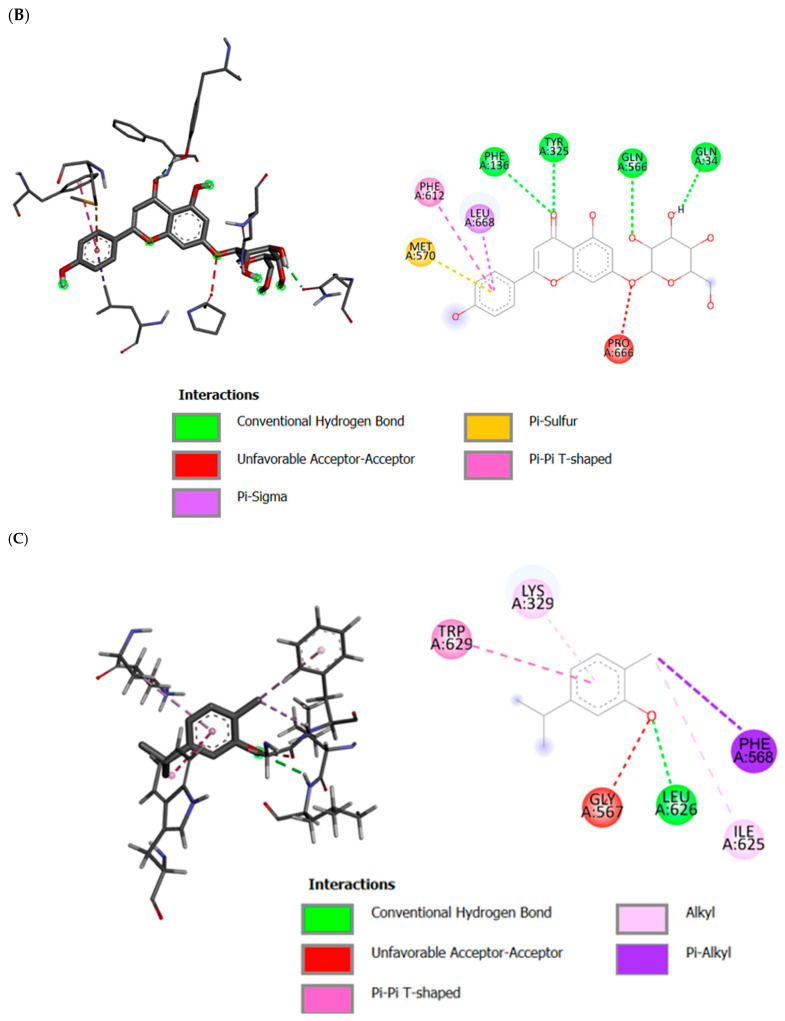

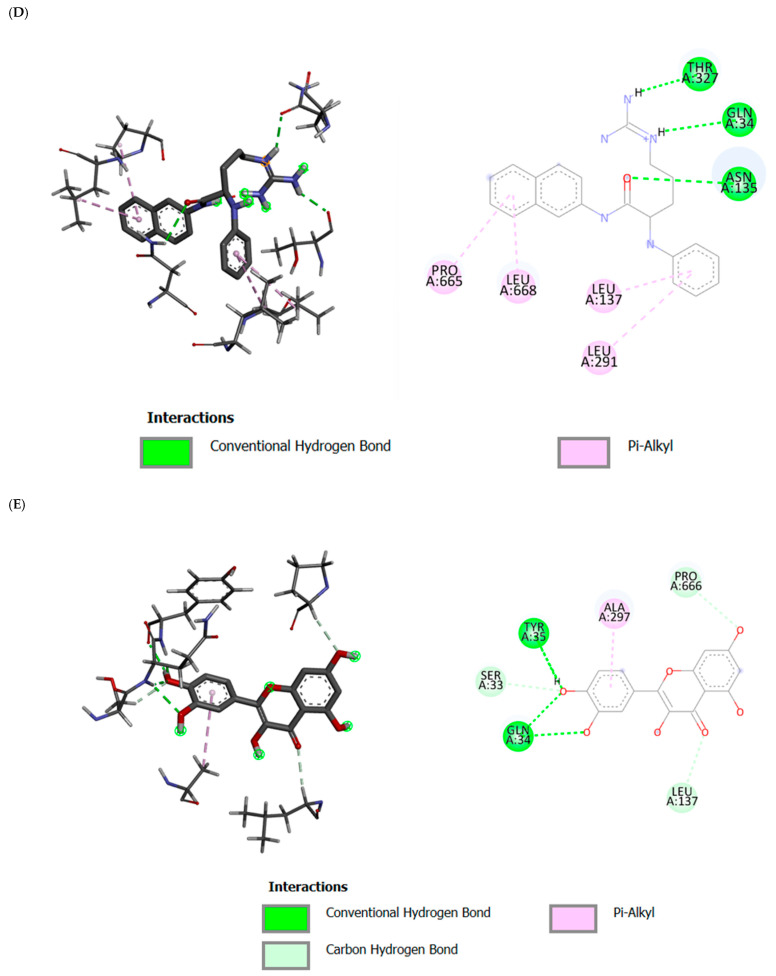
Two-dimensional and three-dimensional interaction network between the MtrD (PDB ID: 6VKS) EP protein in *N. gonorrhoeae* and test compounds from *H. populifolium* generated after molecular docking. As the binding of these compounds within the MtrD efflux pump multi-drug-binding site was within the −5 to −15 kcal/mol an indication that these compounds can fit perfectly within the binding pockets of the MtrD multi-drug-binding site. Hydrogen bonds are noted by the green lines, pi–alkyl interactions by light-pink color, pi–pi T-shaped interaction with the dark pink color, pi–sulfur interaction with the orange color, and unfavorable donor-to-donor interactions with the red color. (**A**) 4,5-dicaffeoylquinic acid. (**B**) Apigenin-7-glucoside. (**C**) Carvacrol. (**D**) Phenylalanine-arginine-β-naphylamide (PaβN). (**E**) Quercetin.

The interaction between the MtrD (PDB ID 6VKS) protein and 4,5-dicaffeoylquinic acid (Figure 2A) displayed a conventional hydrogen bond interaction with SER37, PHE136, GLY295, and ALA 297. A pi–carbon interaction was observed with ARG 133 and PHE612, while GLN 34 revealed unfavorable donor–donor interaction (Figure 2A). Interaction of MtrD EP and apigenin-7-glucoside displayed a classical hydrogen bond interaction network with GLN34, PHE136, TYR325, and GLN566 (Figure 2B). Additional pi–pi T-shaped interactions were revealed with PHE612, pi–sigma interactions with LEU668, and pi–sulfur interactions with MET570 (Figure 2B). The interaction of MtrD EP protein with carvacrol revealed a conventional hydrogen bond interaction with LEU626 (Figure 2C). Additional pi–pi T-shaped interactions were observed with TRP629, pi–alkyl interactions with LYS329, PHE568, and ILE625, and pi–pi T-shaped interactions with TRP629 (Figure 2C). PaβN displayed a conventional hydrogen bond interaction with GLN34, ASN135, and THR327, while pi–alkyl interactions were observed with LEU137, LEU 291, PRO665, and LEU668 residues (Figure 2D). Quercetin interaction networks displayed hydrogen bonds with GLN34 and TYR35 residues. Carbon–hydrogen bond interactions were observed with SER33, LEU137, and PRO666 residues, while pi–alkyl interactions were observed with ALA 297 residue (Figure 2E).

### 2.3. Binding Free Energy Calculations Using the MM/GBSA Approach

To evaluate the strength of the protein–ligand complex formed during molecular docking, the binding free energy (BFE) (ΔG_bind_) of each complex formed was calculated using the molecular mechanics with a generalized born and surface area solvation (MM/GBSA) approach. The ΔG_bind_ values represent the BFE of a ligand to the MtrD protein. This value quantifies the strength and stability of the interaction between the ligand and the protein, with more negative ΔG_bind_ values indicating stronger, more favorable binding. In drug discovery, a lower (more negative) ΔG_bind_ value suggests that the ligand has a higher affinity for the target protein, which is desirable when identifying potential drug candidates. Based on the results (Table 2), the total BFE (ΔG_bind_) computed for 4,5-dicaffeoylquinic acid (INH1), apigenin-7-glucoside (INH2), and carvacrol (INH3) after interaction with MtrD were −54.82 kcal/mol, −47.93 kcal/mol and −16.69 kcal/mol. The positive controls, PaβN (STD1) and quercetin (STD2), revealed binding free energy (ΔG_bind_) of −40.34 kcal/mol and −34 kcal/mol, respectively. The energy exhibited by the Van der Waals forces (ΔE_vdW_) on Apo-INH1, Apo-INH2, Apo-INH3, Apo-PaβN, and Apo-quercetin complexes was −61.7 kcal/mol, −56.06 kcal/mol, −18.10 kcal/mol, −42.42 kcal/mol, and −46.74 kcal/mol, respectively, while the energy for electrostatic forces (ΔE_elec_) in each complex (Apo-INH1, Apo-INH2, Apo-INH3, Apo-PaβN, and Apo-quercetin) was −8.26 kcal/mol, −33.15 kcal/mol, −9.22 kcal/mol, −29.47 kcal/mol, and −123.17 kcal/mol, respectively (see Table 2). The revealed gas-phase interaction energy (ΔG_gas_) of the complexes Apo-INH1, Apo-INH2, Apo-INH3, Apo-PaβN, and Apo-quercetin was −53.4 kcal/mol, −89.21 kcal/mol, −27.30 kcal/mol, −7.89 kcal/mol, and −169.91 kcal/mol, respectively (Table 2). The generalized born electrostatic solvation energy (E_GB_) in the same order of complex listing is as follows: −6.91 kcal/mol, 47.92 kcal/mol, 13.21 kcal/mol, 37.16 kcal/mol, and 142.13 kcal/mol (Table 2). The non-polar solvation energy (E_SA_) produced by the complexes in the same order is as follows: −8.29 kcal/mol, −6.64 kcal/mol, −2.60 kcal/mol, −5.61 kcal/mol, and −6.38 kcal/mol (Table 2). The solvation-free energy (ΔG_sol_) of the complexes was as follows: −1.38 kcal/mol, 41.28 kcal/mol, 10.61 kcal/mol, 31.55 kcal/mol, and 135.75 kcal/mol (Table 2).

### 2.4. Per-Residue Energy Decomposition (PRED) to the Overall Binding Free Energy

**Figure 3:**
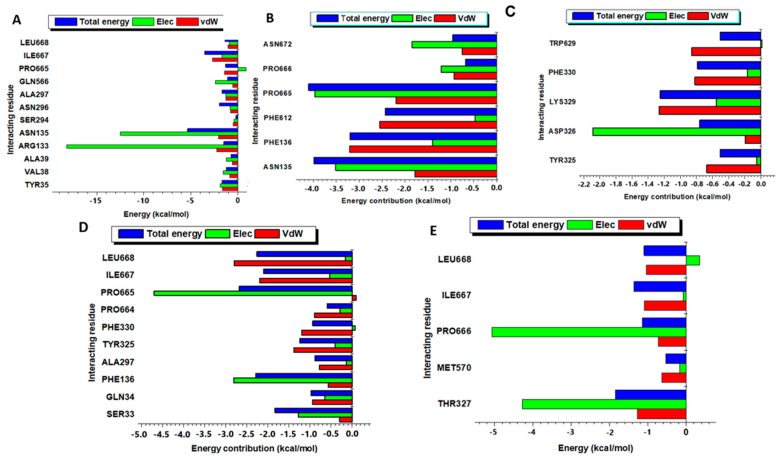
Per-residue energy contributions of the interacting residues of the MtrD protein (PDB ID: 6VKS) with the *H. populifolium* test compounds and standards. (**A**) 4,5-dicaffeoylquinic acid. (**B**) Apigenin-7-glucoside. (**C**) Carvacrol. (**D**) Phenylalanine-arginine-β-naphylamide (PaβN). (**E**) Quercetin.

Per-residue energy contribution evaluates the amount of energy each residue contributed to form the additional interactions in a protein–ligand complex, which includes Van der Waals and electrostatic interactions. The residue contribution in each interaction (electrostatic and Van der Waals interactions), exhibited by the MtrD protein when bound to the test compounds, is shown in Figure 3. From the results, the MtrD-4,5-dicaffeoylquinic acid complex (Apo-INH1) revealed the electrostatic interactions (green bars in Figure 3A) to be contributed by the following residues and energies arginine (ARG 133) (−20 kcal/mol), asparagine (ASN 135) (−13.1 kcal/mol), isoleucine (ILE 667) (−3.2 kcal/mol), ASN 297 (−2.3 kcal/mol), ASN 296 (−2.0 kcal/mol), leucine (LEU 668) (−1.1 kcal/mol), glutamine (GLN 566) (−1.0 kcal/mol), proline (PRO 665) (−1.0 kcal/mol). The Van der Waals forces (red bars, Figure 3A) were contributed by the following residues and their energies: ASN 135 (−7.8 kcal/mol), tyrosine (TYR 32) (−1.5 kcal/mol), arginine (ARG 133) (−1.3 kcal/mol), valine (VAL 368) (−1.1 kcal/mol), and isoleucine 4(ILE 667) (−4.0 kcal/mol). ASN 135 exerted the most energy in this complex of −6.1 kcal/mol (blue bar, Figure 3A).

During the binding of the MtrD protein (PDB ID: 6VKS) and apigenin-7-glucoside (Apo-INH2 complex), the electrostatic interactions (green bars, Figure 3B) were formed by PRO 665 (−4.4 kcal/mol), ASN 136 (−4.0 kcal/mol), ASN 136 (−3.6 kcal/mol), PHE 136 (−3.1 kcal/mol), ASN 672 (−1.8 kcal/mol), PRO 666 (−1.2 kcal/mol), phenylalanine (PHE 612) (−0.3 kcal/mol), and PHE 612 (−0.3 kcal/mol). The Van der Waals interactions (red bars, Figure 3B) were mostly contributed by the PRO 665, PHE 612, PHE 136, and ASN 136 residues. These residues contributed the following energies: PHE 136 (−3.5 kcal/mol), PHE 612 (−2.5 kcal/mol), PRO 665 (−2.1 kcal/mol), and ASN 136 (−1.8 kcal/mol). The overall total energy was contributed mostly by PRO 665 (−4.1 kcal/mol) and ASN 135 (−4.0 kcal/mol) (blue bar, Figure 3B).

Observable interactions between MtrD protein with carvacrol (Apo-INH3) showed electrostatic forces contributed by aspartate (ASP 326) (−2.1 kcal/mol), LYS 329 (−0.5 kcal/mol), and PHE 330 (−0.2 kcal/mol) (Figure 3C, green bars). LYS 329 (−1.3 kcal/mol), tryptophan (TRP 629) (−0.8 kcal/mol), PHE 330 (−0.7 kcal/mol), TYR 325 (−0.65 kcal/mol), and ASP 326 (−0.2 kcal/mol) contributed Van der Waals forces. LYS 329 contributed the highest total energy of all the residues (−1.2 kcal/mol) in this complex (blue bar, Figure 3C).

When PaβN was bound to the MtrD protein (Apo-STD1), noticeable residues contributing to the electrostatic interactions (green bars, Figure 3D) and their energy included PRO 665 (−5.0 kcal/mol), PHE 136 (−2.8 kcal/mol), and (SER 33) (−1.4 kcal/mol) (Figure 3D). On the other hand, LEU 668 (−2.6 kcal/mol), ILE 667 (−2.1 kcal/mol), TYR 325 (−1.5 kcal/mol), PHE 330 (−1.2 kcal/mol), and GLN (−1.0 kcal/mol) (red bars, Figure 3D) were the residues demonstrating noticeable Van der Waals forces. PRO 665 residue contributed the most total energy (−2.5 kcal/mol) of all the interacting residues during the MtrD–PaβN interaction (Apo-STD1) (blue bar, Figure 3D). With the MtrD-quercetin interaction (Apo-STD2), the electrostatic interactions (green bars, Figure 3E) were contributed mainly by PRO 666 (−5.0 kcal/mol) and THR 327 (−4.2 kcal/mol). In contrast, the Van der Waals interactions (red bar, Figure 3E) were mainly contributed by THR 327 (−2.0 kcal/mol) and ILE 667 (−1.1 kcal/mol). LEU 668 (−1.0 kcal/mol) displayed the most total energy compared to all the interacting residues in this complex (blue bar, Figure 3E). Overall, the electrostatic and Van der Waals interactions in each complex contributed to the BFE formed within complexes, a parameter used to understand biomolecular interactions in protein–ligand complexes, thereby facilitating drug discovery.

### 2.5. Dynamic Conformational Stability and Fluctuation of the Studied Compounds

#### 2.5.1. Conformational Stability of the Studied Compounds

**Figure 4:**
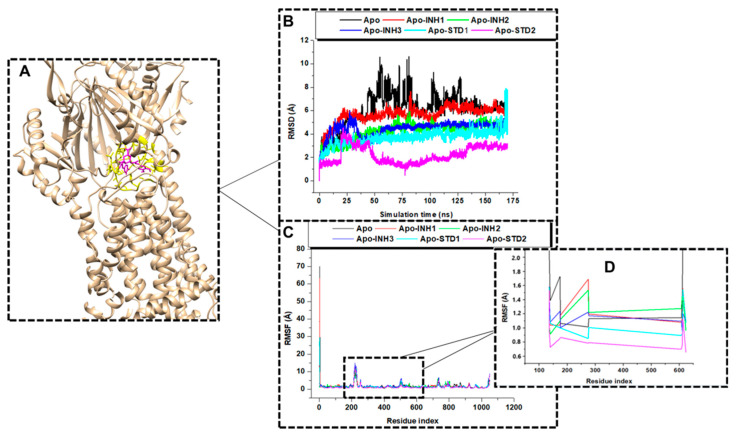
(**A**) Protein–ligand complex of the MtrD efflux pump bound by 4,5-dicaffeoylquinic acid in magenta. The yellow color on the MtrCDE efflux pump protein structure represents the proximal and distal multi-drug efflux pump residues interacting with 4,5-dicaffeoylquinic acid. (**B**) Comparative C-α RMSD plots displaying the degree of stability and convergence of the studied compounds when bound to the MtrD protein for a period of 175 ns. (**C**) Comparative C-α RMSF plot shows the degree of flexibility of the studied compounds when bound to the MtrD protein for a period of 175 ns. (**D**) Comparative C-α RMSF plot, showing the degree of flexibility of the studied compounds when bound to the drug-binding site for a period of 175 ns. For both RMSD and RMSF plots, the black line graph represents data for the Apo protein (Unbound MtrD protein), the red line graph shows Apo-INH1, the green line graph shows Apo-INH2, the dark blue line graph shows Apo-INH3, the light blue line graph shows Apo-STD1, and the pink line graph shows Apo-STD2.

An additional examination of the interaction between the evaluated compounds and the MtrD protein as displayed in Figure 4A was undertaken to assess the complex’s stability. By ensuring the stability of the system, we aimed to identify any perturbed motions and mitigate potential simulation artifacts. In this investigation, the root mean square deviation (RMSD) was computed for the bound and unbound systems to evaluate the system’s stability over a 175 ns duration when in complex with the compounds. The resulting average RMSD values were plotted as shown in Figure 4B. Apo protein revealed an average RMSD score of 6. 10 Å (see black line graph in Figure 4B). The MtrD protein interaction with the studied compounds revealed the following average RMSD scores: 5.65 Å for 4,5-dicaffeoylquinic acid (INH1, see red line graph Figure 4B) and 4.45 Å for apigenin-7-glucoside (INH2, see green line graph in Figure 4B) and 4.24 Å carvacrol (INH3, see dark blue line graph in Figure 4B). The positive controls PaβN (STD1) (light blue line graph, see Figure 4B) revealed an RMSD score of 3.71 Å, while quercetin (STD 2) (pink line graph, see Figure 4B) revealed an RMSD value of 2.22 Å. The observed RMSD of the bound proteins showed no significant deviations from the unbound protein, except for the reference drugs (STD1 and STD2) displayed. Overall, the system was more stable when bound to the evaluated compounds than when unbound, an indication of potential EPI activity by the test compounds. The order of increasing stability was as follows: Apo < Apo-INH1 < Apo-INH2 < Apo-INH3 < Apo-STD1 < Apo-STD2, where from this, we can deduce that carvacrol displayed more stable interactions with the MtrD protein due to the lowest average RMSD score obtained. This was followed by apigenin-glucoside, while 4,5-diaffeoylquinic acid revealed the least stability.

#### 2.5.2. Dynamic Conformational Fluctuations of the Analyzed Compounds

The root mean square fluctuation (RMSF) of the C-α backbone for the Apo system and the bound systems were monitored over 175 ns simulation time, an additional parameter to estimate MtrD protein fluctuations, after interacting with the test compounds (Figure 4C). Apo (unbound MtrD protein) revealed an average RMSF score of 1.936 Å. The complexes Apo-INH1, Apo-INH2, Apo-INH3, Apo-STD1, and Apo-STD2 revealed average RMSF scores of 1.877 Å, 1.644 Å, 1.837 Å, 1.638 Å, and 1.340 Å, respectively. To further gain insight into the fluctuations of the MtrD protein residues located in the multi-drug-binding site of the MtrD protein, the average RMSF scores on the multi-drug-binding site were computed (Figure 4D). The complexes Apo-INH1, Apo-INH2, Apo-INH3, Apo-STD1, and Apo-STD2 revealed average RMSF scores of 1.230 Å, 1.187 Å, 1.186 Å, 1.107 Å, and 0.884 Å (Figure 4D), respectively. Interaction of the studied compounds with the MtrD protein on the multi-drug-binding site revealed fewer fluctuations of the MtrD amino acid residues than the amino acid fluctuations observed at any other part of the MtrD. The overall order of decreasing fluctuations after analysis of interaction on the drug-binding site was as follows: Apo < Apo-INH1 < Apo-INH2 < Apo-INH3 < Apo-STD1 < Apo-STD2. Based on the RMSF scores, carvacrol was revealed to be the most common compound to exert fewer fluctuations when bound to the MtrD protein compared to the other test compounds (4,5-dicaffeoylquinic acid and apigenin-7-glucoside).

### 2.6. Pharmacokinetics and Pharmacochemical Evaluation of the Studied Compounds

After evaluating the studied compound’s stability and fluctuation while in complex with the MtrD protein, compounds were further evaluated for their drug-likeness properties using the SwissADME web server. All studied compounds obeyed the Lipinski rule of five except for 4,5-dicaffeoylquinic acid, which displayed three violations of the rule, including molecular weight (MW) > 500 Da, NorO > 10, and NHorOH > 5 (see Table 3). The compound 4,5-dicaffeoylquinic acid displayed the highest number of heavy atoms which was thirty-seven (37), while carvacrol displayed the lowest number of heavy atoms which was eleven (11). Apigenin-7-glucoside and 4,5-dicaffeoylquinic acid displayed more than 10 hydrogen bond acceptors, while the rest of the compounds displayed less than 10 hydrogen bond acceptors (Table 3). All compounds displayed a molar refractivity greater than 100 except for carvacrol and quercetin. All compounds displayed TPSA ≥ 131.36 Å^2^, except for carvacrol, which displayed TPSA of 20.23 Å^2^, respectively. The log Po/w value of all the compounds was <3.0.

In the same light, the Log S (E_sol_ and SILICOS-IT) parameter was also assessed on the compounds, and all the compounds displayed solubility (Table 3). Of the evaluated compounds, carvacrol showed the ability to permeate the blood-brain barrier (BBB) with Log Kp ≥ −4 cm/s. Both the 4,5-dicaffeoylquinic acid and apigenin-7-glucose were predicted as non-inhibitors of CYP1A2, CYP2C19, CYP2C9, CYP2D6, and CYP3A4.

Quercetin and carvacrol could not inhibit CYP1A2. PaβN is a non-inhibitor of CYP1A2 and CYP3A4. Quercetin was predicted as a non-inhibitor of CYP2C19 and CYP2C9. Only 4,5-dicaffeoylquinic acid and apigenin-7-glucoside were displayed to be P-glycoprotein substrates. PaβN obeyed the Ghosem Veber, Eagen, and Muegge rules, while 4,5-dicaffeoylquinic acid disobeyed all of them. Carvacrol obeyed the Vebber and Eagen, while apigenin-7-glucoside and PaβN only obeyed the Ghose rule. Quercetin and PaβN scaled through the lead-likeness test. Moreover, carvacrol, apigenin-7-glucoside, and PaβN had 0 alert PAINS, while 4,5-dicaffeoylquinic acid and quercetin displayed one alert due to the presence of the catechol A compound on the structure (Table 3).

### 2.7. Validation of H. populifolium Compound’s Antibacterial Activity Against N. gonorrhoeae ATCC 49981

The antibacterial activity of the studied compounds was evaluated before subjecting the compounds to an in vitro efflux pump inhibition assay. This was to understand the baseline antibacterial activity of the phytochemical compounds, ensuring that they possess limited to no bactericidal activity at the concentrations used. Obtained observations suggested 4,5-dicaffeoylquinic acid and carvacrol with the best MIC of 0.125 mg/mL, while apigenin-7-glucoside acid revealed a MIC of 0.250 mg/mL (see Table 4). The positive control, phenylalanine-arginine-β-naphylamide, and quercetin displayed significant MIC values of 0.01 mg/mL and 0.063 mg/mL, respectively. In contrast, the negative control showed no inhibitory activity against *N. gonorrhoeae* ATCC 49981, as the bacterial cells were not subjected to any compound (see Table 4).

### 2.8. Hoechest (Bis-Benzimide) Accumulation Assay

**Figure 5:**
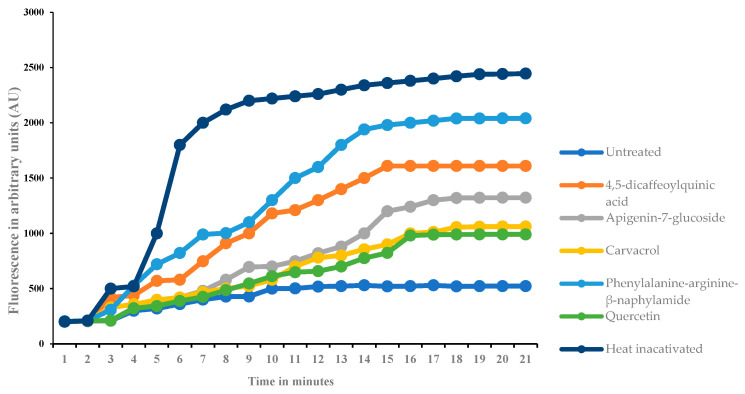
Hoechest (bis-benzimide) accumulation assay in *Neisseria gonorrhoea* ATCC 49981 after treatment with *H. populifolium* compounds: 4,5-dicaffeoylquinic acid (orange line graph), apigenin-7-glucoside (gray line graph), and carvacrol (yellow line graph). PaβN was used as the positive control, while untreated *N. gonorrhoeae* was used as the negative control. The heat-inactivated *N. gonorrhoeae* ATCC 49981 cells serve as a point of reference for maximal dye accumulation due to the loss of membrane integrity, leading to a high influx and retention of the dye.

To validate the potential inhibition of the MtrCDE efflux pump observed in silico, an in vitro study was conducted to evaluate the effects of the test compounds on the *N. gonorrhoeae* efflux pump. The quantitative MtrCDE efflux pump inhibition analysis was conducted using the bis-benzimide accumulation assay (see Figure 5). Fluorescence was detected in arbitrary units, a unitless measure of fluorescence intensity relative to a standard. The higher the fluorescence intensity after treatment, the better the treatment is at inhibiting the MtrCDE efflux pump activity. Treatment with the test compounds revealed 4,5-dicaffeoylquinic acid with the highest fluorescence accumulation of 1610 AU (Figure 5, orange line graph), an indication of EPI activity. This was followed by apigenin-7-glucoside with a fluorescence intensity of 1320 AU (see Figure 5, gray line graph), followed by carvacrol with a fluorescence intensity of 1060 AU (Figure 5, yellow graph). Treatment with quercetin revealed a fluorescence intensity of 980 AU (Figure 5, green line graph), a while treatment with PaβN revealed a fluorescence intensity of 2040 AU (Figure 5, light blue line graph). The untreated *N. gonorrhoeae* cells act as a negative control, displaying how these pumps pump out the dye when treatment is not applied, similar to how they pump the antibiotics; hence, the lowest fluorescence intensity of 522 AU was observed (Figure 5, dark blue line graph). Heat-inactivated cells were included in the study, and they demonstrated the maximum accumulation of the dye in the absence of efflux pump activity.

## 3. Discussion

The continuous emergence of the MDR *N. gonorrhoeae* strains presents a huge quandary for the development of its new effective treatments [1]. The incessant expression and activation of multi-drug efflux protein that ejects intracellular drug concentrations, leading to drug ineffectiveness, is one of the major mechanisms of MDR shown by *N. gonorrhoeae* [1]. The MtrCDE protein is highly expressed in *N. gonorrhoeae*, and is documented for enhanced MDR virulence [7]. Loss or inactivation of this MtrCDE protein consequently modulates the pathogen’s susceptibility to antibiotics. In this manner, the efflux pump protein is considered a reliable drug target site when developing drugs for the treatment of gonorrhoea infection [1].

The identification and development of new antimicrobial agents, adjuvants to ineffective antibiotics, or targeting efflux pump proteins, offer promising alternatives to combat MDR pathogens by reviving the efficiency of antibiotics and the susceptibility of pathogens [21,22]. These antimicrobial attempts contribute to the continuous fight against microbial resistance and hold the potential to develop effective treatments against the infection [23]. In this context, the emergence of computational screening of natural compounds has proved to be an excellent tool for identifying potential EPIs and antivirulence properties [17]. Hence, we adopted computational approaches, including MD, MDS, and ADME analysis, to evaluate the potential EPI activity of the test compounds.

MD contributed to assessing the binding affinities between the test compounds and the MtrD protein. The observed findings offered an understanding of the formed MtrD-test compound’s complex interactions. Compounds 4,5-dicaffeoylquinic acid (INH1) and apigenin-7-glucoside (INH2) displayed docking scores of −8.8 kcal/mol and −8.6 kcal/mol, which were comparable to the standard EPI, PaβN (STD1) (−8.2 kcal/mol) (Table 1). The comparable docking scores may be attributed to test compounds being competitive inhibitors of substrate binding to multi-drug efflux pumps, impeding MDR bacteria [24]. The PaβN docking score is congruent to the study by Jain et al. [8], where PaβN exhibited an MD score of −7.9 kcal/mol when interacting with the MtrD protein. The good docking scores in 4,5-dicaffeoylquinic acid (−8.8 kcal/mol), apigenin-7-glucoside (−8.6 kcal/mol), and carvacrol (−6.0 kcal/mol) (see Table 1) suggest that the compounds studied may be highly active physiologically, due to their ability to provide energy and promote interaction between themselves and the efflux pump protein [16]. In addition, these good MD scores may be attributed to the presence of aromatic rings, hydrogen bond donors and acceptors (OH), amino groups (NH_2_), and carbonyl groups present in 4,5-dicaffeoylquinic acid, apigenin-7-glucoside, and carvacrol. This allows them to establish various favorable interactions with the amino acid residues in the binding site of the MtrD protein [25].

Additionally, the noted hydrogen bond interactions are essential in determining the specificity and directionality of a compound bound to a target protein [26]. Amino acid residues that contributed to the hydrogen bonds observed between the test compounds and the MtrD protein interaction were comparable to the amino acid residues that formed hydrogen bonds when PaβN, a known EPI, interacted with MtrD (see Figure 2A–E). PaβN formed a hydrogen bond with the following amino acid residues: GLN 34, ASN 135, and THR 327 (see Figure 2D), while quercetin formed hydrogen bonds with the following amino acid residues: GLN 34, TYR 35, LEU 137, and PRO 666. These hydrogen bond interactions were similar to some of the hydrogen bond interactions formed when the tested compounds interacted with the MtrD protein. For instance, apigenin-7-glucoside formed hydrogen bonds with similar amino acid residues, GLN 34, PHE 136, TYR 325, and GLN 566 (see Figure 2B). 4,5-dicaffeoylqunic acid (see Figure 2A) also revealed similar hydrogen bond interaction with apigenin-7-glucoside exerted by PHE 136 while carvacrol (see Figure 2C) revealed hydrogen bond interaction with LEU 626 residue. This suggests that 4,5-dicaffeoylquinic, apigenin-7-glucoside, and carvacrol may exhibit the same EPI on MtrD protein, as they shared two or more amino acid residues during hydrogen bond formation with PaβN and quercetin on the drug-binding site.

When the protein–ligand complexes formed during molecular docking were further evaluated for their binding affinity, stability, and fluctuation during molecular dynamics simulations (MDS), this provided quantitative estimates of the strength of the complex interactions [27,28]. This allowed for comprehension of the thermodynamics behind the binding of the evaluated ligands to the MtrD protein, which contributes to the overall BFE and prediction of binding affinities [28]. This further aids in guiding drug discovery efforts [29]. The lower the BFE of a compound, the more improved binding capabilities of a compound are observed [27]. The thermodynamic per-residue contributions were also analyzed for the conclusive BFE exhibited by the test compounds to the MtrD protein.

The 4,5-dicaffeoylquinic acid bound to the MtrD protein (Apo-INH1) displayed the strongest binding free energy of −54.82 kcal/mol (Table 2) compared to all the tested compounds and the positive controls. This suggests a good binding interaction between this ligand and the MtrD protein, which correlates to the docking results observed (−8.8 kcal/mol; Table 1), outlining its ability to fit in well with the binding pockets of the MtrD protein [28]. Furthermore, a strong BFE observed is potentially contributed by a combination of the hydrogen bond interactions explained previously, the Van der Waals forces, and the electrostatic interactions formed between the test compound and the MtrD protein.

The following amino acid residues in MtrD further contributed to the noted BFE: asparagine (ASN) 135 (−14.0 kcal/mol) and arginine (ARG) 133 (−20 kcal/mol) (Figure 3A). For the Van der Waals interaction, MtrD protein residues contributed the following energies when interacting with the 4,5-dicaffeoylquinic acid compound: ARG133 (−3.0 kcal/mol) and ILE667 (−3.1 kcal/mol) (Figure 3A). The ability of these residues to form interactions that automatically lead to a lower BFE may be attributed to their chemical properties [30]. For example, arginine is positively charged at physiological pH, making it more capable of forming electrostatic interactions with negatively charged groups on the 4,5-dicaffeoylquinic acid ligand [30]. Additionally, asparagine can participate in hydrogen bonding, a factor that contributes to electrostatic interactions, while isoleucine (ILE) contributes to hydrophobic interactions due to its aliphatic side chain [31].

MtrD-apigenin-7-glucoside complex (Apo-INH2) (see Figure 3B) revealed the second-lowest BFE (−47.93 kcal/mol) (see Table 2) prompting satisfactory binding affinity interaction and potential to modulate the MtrD efflux pump. The observed BFE is contributed by a combination of the hydrogen bonds, Van der Waals forces, and the electrostatic interactions [32]. Here, the Van der Waals interactions (consult red bars in Figure 3B) were contributed by residues PRO 665, PHE 612, PHE 136, and ASN 136. Proline (PRO) residue consists of a cyclic structure in its side chain backbone, which helps PRO to engage in Van der Waals interactions [32]. Additionally, PRO has the potential to participate in hydrophobic interactions due to its relatively non-polar nature, which further contributes to Van der Waals within the protein structure [32,33]. Aspartate (ASP) residue was able to form Van der Waals interaction with apigenin-7-glucoside. This may have been attributed to the aliphatic portion of the aspartate residue side chain [27]. Phenylalanine (PHE) enhanced Van der Waals interactions when interacting with apigenin-7-glucoside, due to its aromatic ring [31]. PRO666, ASN 136, and ASN 672 contributed the most electrostatic force (consult green bars in Figure 3B). PRO 666 residue has been reported to contribute to electrostatic interactions indirectly due to its lack of charged side chains. However, its contribution is currently being investigated [34]. Asparagine (ASN) is known to consist of a polar uncharged amide group that contributes to electrostatic interactions through hydrogen bonding [30], where, as a result, electrostatic interactions with apigenin-7-glucoside were observed.

Overall, the BFE of −16.69 kcal/mol (Table 2) with MtrD–carvacrol complex suggests a reasonably good binding affinity between MtrD and carvacrol. However, compared to 4,5-dicaffeoylquinic acid (INH1) and apigenin-7-glucoside (INH2), carvacrol exhibited a much weaker binding affinity. The Van der Waals forces that contributed to the overall BFE were formed by the interaction of LYS 329 (−1.3 kcal/mol), TRP 629 (−0.9 kcal/mol), PHE 330 (−0.8 kcal/mol), and TYR 325 (−0.7 kcal/mol) residues (see Figure 3C) with MtrD. The chemical structures of these amino acid residues, such as phenylalanine (PHE) and tyrosine (TYR), consist of an aromatic group in their side chain, which forms Van der Waals forces through dispersion forces [32]. Tryptophan (TRP), on the other hand, consists of an aromatic side chain with a nitrogen atom. This indole group contributes to the Van der Waals interaction due to its bulky nature and the presence of pi–electron clouds [35]. Electrostatic forces also played a role in the overall BFE, and this was evident due to ASP 326 (−2.1 kcal/mol), LYS 329 (−0.5 kcal/mol), and PHE 330 (−0.2 kcal/mol) (Figure 3C). ASP is negatively charged at physiological pH due to the carboxyl (COO^-^) group, which contributes to electrostatic interactions with the carvacrol [30]. Lysine has a positively charged side chain at physiological pH due to the presence of the amino group, which enables electrostatic interactions such as salt bridges with negatively charged groups in the ligand [30].

The root mean square deviation (RMSD) and root mean square fluctuation (RMSF) provide data on the stability of the protein–ligand complex and the fluctuation of the target protein amino acid residues upon binding of the ligand during molecular dynamics simulation [16]. The stability of a protein–ligand complex is crucial, as it correlates to the efficacy of the drug [9,16,26]. This is because compounds with high stability and less fluctuation in complex with the MtrD protein would be more likely to exert their intended pharmacological effects by effectively modulating the protein’s function [33,36]. The RMSD and RMSF analysis mimics the behavior of biological molecules within nanoseconds to microseconds, making it more reliable to quantify the stability of the evaluated protein–ligand complexes [24,37]. The lower the RMSD score, the greater the improvement in the stability of the protein–ligand complex, as this implies that there is less deviation of MtrD amino acid residues upon binding of the compounds [16,36]. The lower the RMSF, the lower the fluctuation of amino acid residues of the target protein when bound to the ligands [16,36].

Based on the results, when the test compounds were in complex with the MtrD protein, low RMSD scores were observed, compared to the RMSD scores observed when the MtrD protein was unbound to a ligand (see Figure 4B). The complex formed when carvacrol (INH3) bound to the MtrD protein resulted in less deviation of MtrD amino acid residues from the reference, as it revealed a lower RMSD score (3.98 Å) compared to other test compounds (see Figure 4B). This suggests that carvacrol was bound to the MtrD protein for a longer period, without falling, dwindling, or dropping off throughout the simulation period, increasing its chances of exerting pharmacological properties on the protein. The positive controls, PaβN (STD1) (2.22 Å) and quercetin (STD2) (3.71 Å), displayed the lowest average RMSD scores (see Figure 4B). This implies that the two compounds bind stably to the MtrD protein throughout the simulation with minimal movement away from its binding conformation [19,20].

The low average RMSD scores observed after compounds interacted with the MtrD protein highlight the studied compounds as potential drug candidates with potential EPI activity due to the low deviation of the amino acid residues of the MtrD protein after interaction with the compounds compared to when it was unbound. Low deviation of amino acid residues in the target protein after binding is directly related to the biological activity of the compounds, whereby if the interaction is unstable/exerts a significant deviation, the compound may not exert its desired effects on the protein, based on factors such as falling off from the bound protein [35,36,37]. Additionally, stable interactions ensure that the drug binds to the target protein consistently and with sufficient affinity [28].

The root mean square fluctuation (RMSF) parameter, on the other hand, measured the displacement of atoms relative to the reference structure and averaged across the number of atoms [36]. We, therefore, computed the average RMSF to elucidate the flexibility and motion of individual residues on the whole protein system and on the multi-drug-binding site when test compounds interacted with the MtrD protein. Computing the average RMSF for the whole system (see Figure 4C) allowed for the assessment of the overall flexibility of the system, with high RMSF values indicating regions that are more flexible and dynamic after binding of the compounds to the MtrD protein, while low RMSF scores display less flexible regions [16,33]. Similarly, analyzing the average RMSF scores for the multi-drug-binding site (see Figure 4D) allowed for the identification of amino acid residues in the MtrD protein that exhibit significant fluctuations in the multi-drug-binding site region, a phenomenon that is essential in drug design [33]. The average RMSF values of the whole system ranged from 1.94 Å to 1.34 Å (see Figure 4C), while for the multi-drug-binding site, it ranged from 1.230 Å to 0.88 Å (see Figure 4D). However, the RMSF score of the apo, 1.936 Å, indicated a relatively high level of flexibility in the absence of a bound ligand, meaning that the binding of the test compounds reduced the flexibility of the MtrD amino acid residues. In both RMSF assessments, carvacrol revealed the lowest average RMSF scores, with 1.837 Å for the whole protein system analysis (see Figure 4C) and 1.186 Å (see Figure 4D) for analysis of amino acid residues in the multi-drug-binding site. This implies that after the binding of carvacrol on the MtrD efflux pump protein, the MtrD retained a consistent conformation, which is a desirable property in drug design as it suggests that the ligand stayed securely bound without causing excessive flexibility in the protein. The less flexibility observed with carvacrol may be attributable to the compound’s chemical structure, which contains several hydroxyl (-OH) functional groups that form stable hydrogen bonds with the MtrD protein residues [33]. The above suggests that carvacrol may potentially be an effective EPI against the MtrD protein.

MtrD-apigenin-7-glucoside complex (Apo-INH2) revealed the second-lowest average RMSF score (1.644 Å) for the entire protein (see Figure 4C) system and (1.187 Å) for the multi-drug-binding site (see Figure 4D). This implies that the binding of apigenin-7-glucoside (INH2) to MtrD does not impose large conformational changes during simulation. This is a good trait when evaluating drug interactions, as massive structural changes in the protein after binding the ligand might either affect the protein or weaken the interaction of the protein with the ligand. Additionally, apigenin-7-glucoside is a flavonoid. Flavonoids have been documented to possess EPI activity based on their polyphenolic structures that allow them to interact with various cellular components, in this case, the MtrD protein, due to their ability to form hydrogen bonds and hydrophobic interactions [38].

Although Apo-INH1 revealed a good docking score, it had a relatively high average RMSF score for the whole system (1.877 Å) (Figure 4C) and the drug-binding site (1.230 Å) (Figure 4D) when compared to the other test compounds. However, the binding of the 4,5-dicaffeoylquinic acid to the MtrD protein dropped the average RMSF score to 1.877 Å, meaning that this binding reduced the flexibility of the amino acid residues in MtrD protein, but not to a very large extent. This also suggests that the 4,5-dicaffeoylquinic acid interactions predicted by molecular docking were insufficient to maintain a stable Apo-INH1 complex in a dynamic environment. This may be due to the MtrD protein or the ligand itself undergoing conformational changes during simulations that disrupt the initial binding interactions over time. However, this makes 4,5-dicaffeoylquinic acid a good candidate for optimization to enhance its stable interactions with the MtrD protein and improve its potential as a therapeutic agent.

On the other hand, carvacrol (INH3) revealed a lower binding affinity for MtrD compared to the other tested compounds (−6.0 kcal/mol) (Table 1) during molecular docking (MD), however, displayed the lowest RMSD score compared to the other tested compounds (see Figure 4B), which implies, that the binding of carvacrol did not deviate the amino acid residues in MtrD protein so much from the reference. This implies that the MtrD–carvacrol complex remained stable throughout the simulation, with minimal conformational changes. The relatively weak docking score observed when carvacrol interacted with the MtrD protein, compared to apigenin-7-glucoside and 4,5-dicaffeoylquinic acid, may be attributed to the molecular docking principle, which utilizes simplified scoring functions and does not capture the dynamic behavior of the system [28]. Consequently, a high docking score observed with carvacrol may not have accurately reflected the stability of the complex over time, and this was supported by the low average RMSD score observed. Alternatively, both carvacrol and 4,5-dicaffeoylquinic acid may have undergone conformational changes during simulation, leading to improved fitting in the MtrD protein, thereby optimizing interactions between the compound and the protein [28,29]. Otherwise, the positive controls, PaβN revealed a 1.340 Å average RMSF score for the whole system (see Figure 4C) and 0.884 Å for the drug-binding site) (see Figure 4D) while quercetin revealed a 1.638 Å RMSF score for the whole system ((see Figure 4C) and a 1.107 Å RMSF score on the drug-binding site) (see Figure 4D). This suggests good stability of these compounds when bound to the MtrD efflux pump. This observation was expected, as these compounds have been documented to be known EPIs [19,20].

The assessment of the test compounds for physicochemical properties, lipophilicity, water solubility, and pharmacokinetics properties to characterize their drug-likeness properties was conducted to optimize the test compound’s efficacy and safety profiles in drug development [39]. Favorable ADME (absorption, distribution, metabolism, and excretion) properties of the test compounds were evaluated based on the Lipinski rule of five [39,40]. The rule classifies the poor absorption of a drug when there are more than five hydrogen bond donors, more than 10 hydrogen bond acceptors, greater than a 500 Da molecular weight, and a greater than 5 estimated Log P (CLog P) [40,41]. Based on the results, all compounds obeyed the Lipsinki rule of five, except for 4,5-dicaffeoylquinic acid, which displayed three violations (MW > 500 Da, Nor > 10, and NHorOH > 5) (Table 3). Apigenin-7-glucoside displayed one violation, which is still considered acceptable according to [41,42], while carvacrol displayed no violations (Table 3). This suggests that apigenin-7-glucoside and carvacrol have a higher probability of being orally bioavailable and potentially suitable for further development as drug candidates [39]. To further support carvacrol and apigenin-7-glucoside drug potency, heavy atoms were observed in both compounds. Heavy atoms have been reported to be an essential chemical structure-related character associated with the compound’s drug-likeness properties and physicochemical properties [42].

Pharmacokinetic properties such as the Egan Filter, Ghose Filter, Veber filter, and Muege filter also contributed to the drug-likeness characteristics of the studied test compounds, as displayed in Table 3. Carvacrol displayed the potential to permeate the blood-brain barrier (BBB), which implies its potential to pass through the central nervous system, indicating its therapeutic ability [39,42]. These findings are congruent to a study by Zotti et al. [42], where carvacrol’s ability to permeate the BBB was attributed to the compound’s lower molecular weight of about 150.2 g/mol, its lipophilic structural profile, and possibly due to its broad therapeutic activities which include antibacterial, anti-inflammatory and, antioxidant properties. Compounds, 4,5-dicaffeoylquinic acid, and apigenin-7-glucoside were found to be non-inhibitors of CYP1A2, CYP2C19, CYP2C9, CYP2D6, and CYP3A4 drug metabolism enzymes. For this reason, these two compounds do not potentially interfere with the metabolism of other drugs that are substrates to these enzymes. This is advantageous since it reduces the risk of adverse drug interactions when co-administered with other medications metabolized by these enzymes [43]. This information contributes to determining dose regimens and assessing the therapeutic index of the compounds [43]. All the tested compounds were found to be soluble. High solubility typically correlates with improved bioavailability, where more of the administered dose reaches the systemic circulation, thereby increasing the chances of therapeutic concentrations at target sites [41].

The PAIN (Pan Assay Interference) assay structural alerts in medicinal chemistry are known to predict toxic and unstable fragments on the test compound structures rather than predicting drug-like properties [44]. The alerts in PAIN suggest that the compounds might exhibit undesirable properties that could interfere with experimental assays or potentially lead to false positive or false negative results in biological assays [44]. Apigenin-7-glucoside and carvacrol displayed 0 alerts PAINS, making them promising potential drug candidates that could be used to treat gonorrhoeae infections. The 4,5-dicaffeoylquinic acid compound had one alert (see Table 3), indicative of a motif flagged as a problematic feature. However, it is important to note that the presence of alerts in PAINS does not eliminate the potential of the evaluated compound as a useful drug candidate, as this signals the requirement of additional tests and evaluations to determine the compound’s suitability for further development [45].

The Brenk alerts are associated with structural motifs within chemical compounds that are associated with potential issues in drug discovery such as toxicity, metabolic instability, or interference with biological assays [46]. All test compounds displayed 0 Brenk alerts except for 4,5-dicaffeoylquinic acid, which had one alert (Table 3). This implies a low likelihood of exhibiting problematic properties, more so having zero or one Brenk alerts does not guarantee that the compounds are completely free of undesirable properties. In essence, all the test compounds contain many structural features associated with known issues and would still require a thorough evaluation across various assays to assess their overall suitability as drug candidates. However, the ability of the studied compounds to block the activity of an efflux pump was further evaluated in vitro.

In validating the in silico results, the in vitro efflux pump inhibitory activity of these compounds was conducted. However, before then, the compounds were evaluated for their antibacterial activity. This was done to determine the concentrations of the test compounds that do not kill the bacteria, to ensure that any observed inhibition of the dye during the bis-benzimide accumulation assay, would be due to the compound’s effect on the pump rather than bacterial death [47]. The minimum inhibitory concentration assay was conducted for the evaluation of the antibacterial activity of the compounds. Results ranged from 0.125 to 0.25 mg/mL after treatment with 4,5-dicaffeoylquinic acid and carvacrol, displaying the most potent MIC value of 0.125 mg/mL, a notably lower threshold when compared to apigenin-7-glucoside. This may be attributed to 4,5-dicaffeoylquinic being a quinic acid derivative, as these derivatives are known to possess antibacterial properties due to their ability to disrupt cell membranes, inhibit bacterial enzymes, and interfere with cellular processes [47]. Carvacrol’s antibacterial activity is known to revolve around its hydrophobic nature caused by the presence of hydroxyl groups attached to benzene rings, facilitating interaction with the bacterial cytoplasmic membrane [48]. It inserts itself between fatty acids within the membrane, inducing expansion and destabilization of the membrane structure, resulting in bacterial death because the membrane is essential for maintaining vital functions such as structural integrity, transport of nutrients and waste, and production of energy [48]. The low antibacterial activity observed with apigenin-7-glucoside may have been attributed, at least in part, to *N. gonorrhoeae* being a Gram-negative pathogen. Gram-negative bacteria possess an outer membrane and murein component in their cell wall, creating a formidable barrier against the penetration of the test compounds [49,50]. Overall, the compounds displayed better antibacterial activity than the positive control quercetin but not with ciprofloxacin. This may be attributed to the test compounds and quercetin being natural plant-based compounds and, therefore, having less specific mechanisms, making their activity less potent compared to synthetic antibiotics, which are optimized for high potency and specificity against bacterial targets.

After the evaluation of compounds for antibacterial activity, quantitative EPI was conducted using the bis-benzimide accumulation assay (Figure 5). The bis-benzimide dye was specifically used in this assay due to it being a known substrate for the MtrCDE efflux pump in *N. gonorrhoeae* [51]. From the results, 4,5-dicaffeoylquinic acid emerged as the most effective compound in reducing the activity of the MtrCDE efflux pump, as evidenced by the high accumulation of the dye within the cell (Figure 5, Orange line graph). This indicates that after treatment, the dye continued to enter the cell, but the pathogen was unable to expel the dye, resulting in its accumulation, demonstrating the compound’s potential efflux pump inhibitory activity. Congruent to this study, Sobhanipoor et al. [52] also documented 4,5-dicaffeoylquinic acid as a good EPI agent in *Staphylococcus aureus* and *Enterococcus faecalis* as it revealed the most efflux pump activity reduction of NorB in *S. aureus* and EfrAB in *E. faecalis* among the tested compounds. The bis-benzimide assay revealed that apigenin-7-glucoside (Figure 5, Gray line graph) had a higher fluorescence intensity than carvacrol (Figure 5, yellow line graph). However, the difference was not statistically significant. This suggests that apigenin-7-glucoside may also be a potential efflux inhibitor. This is also supported by Molina et al. [50], as they confirmed apigenin-7-glucoside-reported EPI activity to be attributed to the compound being a flavonoid. Flavonoids have been reported to possess EPI activity in addition to their antibacterial, antioxidant, and anti-inflammatory pharmacological properties [49].

Additionally, flavonoids act as defense mechanisms against other microbes, which explains the displayed EPI activity and antibacterial activity [49]. Janosity et al. [15] documented carvacrol as an EPI, whereby it acted as a competitive inhibitor of the NorA efflux pump in *S. aureus*, confirming its observed EPI activity in this study. The discovery of potential EPI after treatment with *H. populifolium* compounds in this study suggests these compounds’ potential viability for combination therapy alongside existing antibiotics in combatting gonorrhoea infection. This strategy ensures that antibiotics reach their intended targets without being prematurely expelled, allowing them to remain effective within cells until they eradicate the bacteria. Consequently, this approach not only underscores the importance of developing new drugs but also advocates for the utilization of existing antibiotics, even those previously deemed ineffective due to pathogen resistance, thereby conserving valuable resources used in manufacturing new drugs.

## 4. Materials and Methods

### 4.1. Molecular Docking

#### 4.1.1. Protein Generation and Preparation

The Cryogenic Electron Microscopy (Cryo-EM) structure of MtrD in complex with ampicillin (PDB ID: 6VKS, resolution of 3.02 Å) was retrieved and downloaded from the Research Collaboratory for Structural Bioinformatics Protein Data Bank (RCSB PDB). The downloaded protein was viewed and prepared using UCSF Chimera software package 1.17.1, developed by the Resource for Biocomputing, Visualization, and Informatics, University of California, San Francisco (UCSF), CA, USA [53]. The co-ligands phosphatidylethanolamine (PTY), ampicillin, and water molecules were removed from the crystal structure, and an appropriate number of polar hydrogen atoms were added to the efflux pump. Gasteiger charges were assigned to the target protein, and the non-polar hydrogen atoms were merged into carbon atoms.

#### 4.1.2. Ligand Generation and Preparation

A list of bioactive compounds from *Helichrysum populifolium* was obtained from the literature [13,14,15]. These compounds were selected based on displaying efflux pump inhibition after qualitative and quantitative efflux pump inhibition assessment during in vitro studies. Phenylalanine-arginine-β-naphylamide (PaβN) and quercetin were included in this study as positive controls (reference standards) and for results comparisons. The 2D structures of the ligands were retrieved from the NCBI PubChem database. The 3D conformers of the ligands were generated using LigPrep under the Schrodinger Maestro tool 11.5, LLC, New York, NY,2017.

#### 4.1.3. Ligand Docking

Molecular docking was employed using the Autodock Vina Plugin available on Chimera [53]. The compounds were docked into the conserved proximal and distal drug-binding sites situated not far from each other within the pump. According to Lyu et al. [7], the conserved proximal multi-drug drug-binding site consists of these residues: S79, S134, M570, Q574, F612, E669, L670, G671, R714, G717, and E823. The conserved proximal multi-drug site consists of S134, F136, L175, F176, E271, Y325, M570, V607, F610, F612, and F623. The co-crystalized ligand, ampicillin, displayed interaction with the residues in the distal drug-binding site [7]. However, to maximize the chance of identifying potential EPI that targets multiple regions of the multi-drug-binding site, it was ideal to use both the proximal and distal drug-binding sites. Both the proximal and distal multi-drug-binding sites were selected and docked in this study as they also share similar residues, indicating that they are situated close to each other within the MtrD efflux pump. The grid box around this site was defined with the following dimensions: 40 Å × 52 Å × 66 Å in AutoDock, covering the protein structure while keeping the center of binding site coordinates at 177.948, 174.879, and 223.45, respectively. Detailed information regarding the grid box and the prepared protein and ligands were contained in a configuration file, a text file extension required for docking calculations in AutoDock. The protein structure was kept rigid, while the ligands were flexible during docking. The lower the molecular docking binding scores, the higher the binding affinity. The MtrD efflux pump protein and the test compounds were subsequently separated and prepared for molecular dynamics simulation.

### 4.2. Molecular Dynamics Simulations

A molecular dynamics simulation (MDS) was conducted using the Amber GPU version of the PMEMD engine provided by the AMBER18 package at the Lengau CHPC (https://www.ambermd.org) (accessed on 10 July 2023) [16]. The MtrD–ligand complexes were described with the AMBER18 FF18SB force field variant [36]. The combined procedures, which include restrained electrostatic potential (RESP) and the General Amber Force Field (GAFF) [16,36] in the antechamber package, were used to obtain atomic partial charges for the ligands. Hydrogen atoms, sodium (Na^+^), or chloride (Cl^−^) counter ions were added to the system for neutralization, depending on the charge state of the complex. The complexes were then suspended within an orthorhombic box of TLP3P water molecules to contain the atoms within 12 Å of any box edges.

The systems were initially minimized for 2500 steps with 500 kcal·molÅ^2^ restraint potential applied. An additional 5000 steps of full minimization were carried out without restraints using the conjugate gradient algorithm. The heating step was conducted in a canonical ensemble condition (NVT) by heating the systems gradually from 0 to 300 K for 5 ps, such that the systems maintained a fixed number of atoms and volumes. Potential harmonic restraint and collision frequency of 1.0 ps^−1^ were imposed with the solute within the systems. The protein systems were equilibrated for 10,000,000 steps for a period of 175 ns, while maintaining a constant operating temperature, T (300 K). The number of atoms and pressure were also kept constant. The total simulation trajectory was 175 ns. The SHAKE algorithm was utilized to construct hydrogen atom bonds. Each simulation’s step size was 2 fs, and the SPFP precision model was used. The simulations occurred simultaneously with the isobaric isothermal ensemble (NPT), with randomized seeding, the constant pressure of 1 bar maintained by Berendsen barostat, pressure-coupling constant of 2 ps, temperature of 300 K and Langevin thermostat with a collision frequency of 1 ps.

#### 4.2.1. Post-Molecular Dynamic Analysis

##### Binding Free Energy Analysis

Binding free energy calculations were carried out using the Molecular Mechanics Generalised-Born Surface Area method (MM/GBSA) [54]. The binding free energy is essential for exploring the binding strength of ligands with proteins thermodynamically [54]. The lower the binding free energy, the greater the strength of the interaction. The MM/GBSA was used to calculate and estimate the binding affinities of the bound system. An average of 10,000 snapshots from 20 ns trajectory formed the basis for calculating the free energy. Moreover, MM/PBSA is mathematically represented as follows:

ΔG_bind_ = G_complex_ − G_protein_ − G_inhibitor_ (1)

ΔG_bind_ = E_gas_ + E_sol_ − TΔS (2)

E_gas_ = E_int_ + E_vdW_ + E_elec_ (3)

G_sol_ = G_GB_ + G_SA_ (4)

G_SA_ = γSASA (5)

where the E_vdW_ and E_elec_ represent the Van der Waals. E_gas_ represents gas-phase energy estimated from the FF18SB force field terms, while E_int_ denotes internal energy. G_sol_ denotes the solvation-free energy and can be decomposed into polar and non-polar contribution states. G_SA_, non-polar solvation energy was determined from the solvent-accessible surface area (SASA) using a water probe radius of 1.4 Å. On the other hand, the polar solvation, G_GB_, is determined by resolving the GB expression. The total entropy is determined by S, while T determines the temperature of the systems. The MM/GBSA method in AMBER 18 was used to obtain the total binding free energy each residue contributed at the MtrD drug-binding site by carrying out per-residue energy decomposition at the atomic level.

##### Dynamic Conformational Stability and Fluctuation of the Protein–Ligand Complex

The system’s coordinates and trajectories were analyzed every 1 ps using PTRAJ, followed by the analysis of the binding free energy, root mean square deviation (RMSD), and root mean square fluctuation (RMSF), utilizing the CPPTRAJ module employed in the AMBER 18 suit. Origin data analysis software v9.1, OriginLab Corporation, Northampton, MA, USA [55], was used to generate all raw data plots. The 3D image of the target protein was generated using the UCSF Chimera software package [53].

### 4.3. Drug-Likeness Properties of the Studied Compounds

The test compound’s pharmacokinetic and physiochemical properties were assessed using the SwissADME web server (http://www.swissadme.ch/index.php) (accessed on 10 July 2023) [16]. The SMILES of the compounds were obtained from the PubChem database. The SMILES were then inserted in the webserver for running to occur so that the predicted parameters could be generated. The hit compounds lipophilicity, water solubility, and medicinal chemistry were determined. The drug-likeness properties/rules, including Lipinski’s, Ghose’s, Veber’s, Egan’s, and Muegge’s rules, were computed in order to further assess the test compound’s potential to be used as drugs for the treatment of gonorrhoea infection.

### 4.4. Chemicals and Compounds Used in the In Vitro Assay

The compounds used in this study were also bought from Sigma-Aldrich (Johannesburg, South Africa) and their Identification IDs are as follows: apigenin-7-glucoside (lot no: 22129), 4,5-dicaffeoylquinic acid (lot no: 26647), carvacrol (STBJ9333), quercetin (lot no: LRAB7760), ciprofloxacin (lot no: 098M4006V) phenylalanine-arginine-β-naphylamide (CAS no: 18905-73-2), and bis-benzimide dye (CAS no: 23491-53-3).

### 4.5. Preparation and Culturing of the Neisseria gonorrhoea ATCC 49981

The *N. gonorrhoeae* strain ATCC49981 strain was used in this study because it is a known clinical strain consisting of the MtrCDE efflux pump [1]. The strain was obtained from Sigma-Aldrich (Johannesburg, South Africa). *Neisseria gonorrhoeae* ATCC 49981 was preserved in glycerol stock at −80 °C. The strain was cultured in LB broth and then incubated at 37 °C for 24 h. The standardized bacterial suspension was adjusted using sterile LB broth to achieve an equivalent of 0.5 McFarland standard. This was maintained for all subsequent assays.

### 4.6. H. populifolium Antibacterial Activity Against N. gonorrhoeae ATCC49981 

The compounds’ minimum inhibitory concentration (MIC) against *Neisseria gonorrhoeae* ATCC 49981 was determined using a microdilution method [56] with slight modification. A volume of 100 µL of Lauria Bertani (LB) broth was transferred into every well of the 96-well microtiter plate and 100 µL of each test compound was transferred into the wells of row A of the microtiter plate together with the negative control (1% DMSO) and positive controls, ciprofloxacin at a starting concentration of 0.01 mg/mL and quercetin at a starting concentration of 0.068 mg/mL. Quercetin and ciprofloxacin were added because they are known antibacterial and anti-virulent agents [20,57]. Additionally, a blank (sterile LB broth) and standardized bacterium (control) were prepared by transferring 200 µL to the wells, respectively. Two-fold serial dilutions were conducted, resulting in decreased concentrations over the range of 0.25–0.0019 mg/mL. Thereafter, 100 µL of the standardized bacterium was added to each well. Plates were then incubated at 37 °C for 24 h. After 24 h of incubation, 40 µL of 0.2 mg/mL of p-iodonitrotetrazolium violet (INT) (Sigma-Aldrich, Johannesburg, South Africa) was added to each well, followed by an additional 1 h of incubation. The INT reaction works by transferring electrons from nicotinamide adenine dinucleotide hydrogen (NADH), a product of the threonine dehydrogenase (TDH) catalyzed reaction, to the tetrazolium dye (INT) [56]. Bacterial TDH catalyzes the NAD-dependent oxidation of threonine to produce 2-amino-3-ketobutyrate and NADH. An electron is then transferred from NADH to INT, resulting in the formation of a pink dye, indicative of bacterial growth [56]. The MIC was identified as the lowest concentration that prevented the formation of the pink color that indicated growth after INT addition. All tests were performed in triplicate.

### 4.7. Bis-Benzimide Accumulation Assay

The potential *N. gonorrhoeae* ATCC 49981 MtrCDE efflux pump inhibition following treatment with *H. populifolium* compounds was quantitatively evaluated using a modified method described by [51,52] with minor modifications. *N. gonorrhoeae* ATCC 49981 was grown in LB broth until OD_600nm_ of 0.6 was reached. The cell pellets were harvested through centrifugation at 6000× *g* for 10 min at room temperature. The supernatant was discarded, and the cell pellet was resuspended in phosphate buffer saline (PBS) (0.1 M, pH 7.2). The OD_600nm_ was adjusted to 0.5 using PBS. Thereafter, 150 µL of the standardized bacterial sample was transferred into sterile black 96-well microtiter plates (Thermo Scientific, Pretoria, South Africa), followed by the addition of the compounds (20 µL). Known efflux pump inhibitors (PaβN) (10 µM) and quercetin (1 mg/mL) were used as positive controls, while the untreated *N. gonorrhoeae* cells were used as the negative control. Heat-inactivated *N. gonorrhoeae* cells were also included as controls. These cells were obtained by boiling the standardized *N. gonorrhoeae* cells at 100 °C for 10 min to denature the MtrCDE efflux pump protein and to disrupt the membrane integrity that supports the efflux pump. Any dye accumulation observed in the boiled cells indicates what happens when the efflux pump is not completely active, giving us a baseline to compare with activity in the live-treated *N. gonorrhoeae* cells with uninterrupted efflux pump protein. A volume of 20 µL of bis-benzimide dye (25 µM) was then added to the bacterial samples containing compounds and standardized bacteria in the black 96-well microtiter plates. All treated and untreated samples were incubated for 30 min. Fluorescence was then read at an excitation and emission wavelength of 355 nm and 460 nm using the FLUOstar Omega fluorometer (Labotec, Midrand, South Africa) every minute for 30 cycles to give out results in arbitrary units (AU). The readings obtained were analyzed using Omega data analysis software v10, PerknElmer, Inc., Waltham, MA, USA. Each sample of compounds was tested three times for statistical analysis.

## 5. Conclusions

In silico modeling provides valuable insights into identifying compounds with EPI activity to aid the search for novel drugs to treat infections. Hence, in this study, we validated the test compounds’ (4,5-dicaffeoylquinic acid, apigenin-7-glucoside, and carvacrol) potential EPI activity against the MtrD protein in *N. gonorrhoeae*. MDS analysis showed that carvacrol exerted the most stability when in complex with the MtrD protein for 175 ns, which may have been attributed to conformation changes of the compound to fit in perfectly into MtrD binding pockets. This made carvacrol, followed by apigenin-7-glucoside, the most promising EPI candidates based on their reasonable binding affinity, sustained stability during MDS, and obedience to the Lipinski rule of five. On the other hand, 4,5-dicaffeoyquinic acid was not completely ruled out as a potential EPI, as it displayed good binding affinity and strong in vitro EPI activity relative to other compounds. However, structural modifications may be explored for this compound for future studies to see how its binding and biological activity may change to better conclude that it is a potential drug with EPI activity. Further analysis, such as in vitro cytotoxicity studies, still needs to be done to confirm the toxicity of these compounds on mammalian cells. Overall, the compounds did show potential to reduce *N. gonorrhoeae* MtrD protein activity, although further exploration is still necessary, such as using these compounds with existing antibiotics to evaluate their synergistic effects to better conclude them as potential drugs with EPI activity.

## Figures and Tables

**Table 1:** Molecular docking results of the *H. populifolium* compounds against the MtrD efflux pump protein multi-drug-binding site in *N. gonorrhoeae*.

Compound Name	Class	Smiles Designation of the Compounds	Docking Scores (kcal/mol)	Amino acid Residue Interaction
4,5-dicaffeoylquinic acid	Polyphenold	C1[C@H]([C@H]([C@@H](C[C@@]1(C(=O)O)O)OC(=O)/C=C/C2=CC(=C(C=C2)O)O)OC(=O)/C=C/C3=CC(=C(C=C3)O)O)O	−8.8	GLN34, SER37, ARG133, PHE136, GLY295, ALA297, PHE612
Apigenin-7-glucoside	Flavonoid	C1=CC(=CC=C1C2=CC(=O)C3=C(C=C(C=C3O2)OC4[C@@H]([C@H]([C@@H]([C@H](O4)CO)O)O)O)O)O	−8.6	GLN34, PHE136, TYR325, GLN566, MET570, GLN566, PHE612, PRO664, LEU668
Carvacrol	Terpenoid	CC1=C(C=C(C=C1)C(C)C)O	−6.0	LYS329, GLY567, PHE568, ILE625, LEU626, TRP629
* PaβN	Peptidomimetic	C1=CC=C(C=C1)N[C@@H](CCCN=C(N)N)C(=O)NC2=CC3=CC=CC=C3C=C2	−8.2	GLN34, ASN135, LEU137, LEU291, THR327, PRO665, LEU668
* Quercetin	Flavonoid	C1=CC(=C(C=C1C2=C(C(=O)C3=C(C=C(C=C3O2)O)O)O)O)O	−8.1	SER33, GLN34, TYR35, LEU137, ALA297, PRO666

Standards—*. ALA—alanine, ASN—asparagine, GLN—glutamine, LEU—leucine, TYR—tyrosine, PRO—proline, THR—threonine, PHE—phenylalanine, MET—methionine, LYS—lysine, SER—Serine, TRP—tryptophan.

**Table 2:** Thermodynamics analysis summary of the MM/GBSA-based binding free energy involving test compounds.

	Energy Composition (kcal/mol)						
Complex	ΔE_vdW_	ΔE_elec_	ΔG_gas_	E_GB_	E_SA_	ΔG_sol_	ΔG_bind_
Apo-INH1	−6.07 ± 4.61	−8.26 ± 2.02	−53.04 ± 14.32	−6.91 ± 11.78	−8.29 ± 0.45	−1.38 ± 11.6	−54.08 ± 6.27
Apo-INH2	−56.06 ± 2.81	−33.15 ± 7.33	−89.21 ± 7.09	47.92 ± 4.74	−6.64 ± 0.22	41.28 ± 4.67	−47.93 ± 4.10
Apo-INH3	−18.10 ± 2.63	−9.22 ± 3.46	−27.30 ± 4.10	13.21 ± 2.20	−2.60 ± 0.32	10.61 ± 2.10	−16.69 ± 3.01
* Apo-STD1	−42.42 ± 3.13	−29.47 ± 5.08	−7.89 ± 5.05	37.16 ± 3.00	−5.61 ± 0.12	31.55 ± 3.01	−40.34 ± 3.05
* Apo-STD2	−46.74 ± 3.13	−123.17 ± 5.08	−169.91 ± 5.05	142.13 ± 3.00	−6.38 ± 0.12	135.75 ± 3.01	−34.16 ± 3.05

* Apo is the MtrD protein structure that is not bound to a ligand. Apo-INH1, Apo-INH2, Apo-INH3, Apo-PaβN, and Apo-quercetin mean that the MtrD is bound to a ligand/inhibitor INH1, INH2, INH3, PaβN, and quercetin. INH stands for potential inhibitor. INH1 denotes 4,5-dicaffeoylquinic acid, INH2 denotes apigenin−7-glucoside, and INH3 denotes carvacrol. STD stands for standards (positive controls). STD1 denotes PaβN, and STD2 denotes quercetin. ΔG_bind_ values represent the binding free energy of a ligand to the MtrD protein. This value quantifies the strength and stability of the interaction between the ligand and the protein, with more negative ΔG_bind_ values indicating stronger, more favorable binding. ΔE_vdW_ is the energy exhibited by the Van der Waals forces within the protein–ligand complex, ΔE_elec_ is the energy exhibited by the electrostatic forces within the protein–ligand complex, ΔG_gas_ is the energy formed by the sum of electrostatic and Van der Waals forces, and ΔG_sol_ is the energy change due to solvent effects including polar solvation energy (E_GB_)—is the contribution from electrostatic interactions with the solvent and non-polar solvation energy (E_SA_), the contribution from the non-polar interaction with the solvent.

**Table 3:** Drug-likeness properties of the tested *H. populidolium* compounds.

	Physico-Chemical Properties				Lipophilicity	Water Solubility		Parmaco-kinetic	Drug-Likeness	Medicinal Chemistry	
Compounds	No. of Heavy Atoms	No. of Rotatable Bonds	No. of H. Bonds Acceptors	No. of H bond donors	Consensus log Po/W	LogS	Class	BBB Permeation	Lipinski	PAINS Alert	Brenk Alert
Carvacrol	11	1	1	1	2.82	−3.31	S	Y	Y-No 0 violation	0 alerts	0 alerts
Apigenin-7-glucoside	31	4	10	6	0.55	−3.78	S	N	Y-1 1 violation: NHorOH > 5	0 alerts	0 alerts
4,5-dicaffeoylquinic acid	37	9	12	7	0.78	−3.65	S	N	N-3 Violations 3 MW > 500, NorO > 10, NHorOH > 5	1 alert: Catechol_A	3 alerts: Catechol_A, michael_acceptor_1, more_than_2_esters
* PaβN	28	9	2	4	2.86	−4.05	MS	N	Y-No 0 violation	0 alerts	2 alerts: imine_1, imine_2
* Quercetin	22	1	7	5	1.23	−3.16	S	N	Y-No 0 violation	1 alert: Catechol_A	1 alert: Catechol

* Standards (Positive controls); S = Soluble, MS = Moderately Soluble; Y = Yes, N = No.

**Table 4:** Minimum inhibitory concentration (MIC) (mg/mL) of the *H. populifolium* compounds against *N. gonorrhoeae* ATCC 49981.

Compounds	*Neisseria gonorrhoea* ATCC 49981
4,5-dicaffeoylquinic acid	0.125
Apigenin-7-glucoside	0.250
Carvacrol	0.125
PaβN	0.124
**Controls**	
Quercetin	0.063
Ciprofloxacin	0.01
1% DMSO	8.00

## Data Availability

The original contributions presented in this study are included in the article. Further inquiries can be directed to the corresponding author(s).

## References

[1] Lim K.Y.L., Mullally C.A., Haese E.C., Kibble E.A., McCluskey N.R., Mikucki E.C., Thai V.C., Stubbs K.A., Sarkar-Tyson M., Kahler C.M. (2021). Anti-virulence therapeutic approaches for *Neisseria gonorrhoeae*. Antibiotics.

[2] Rowley J., Vander Hoorn S., Korenromp E., Low N., Unemo M., Abu-Raddad L.J., Chico R.M., Smolak A., Newman L., Gottlieb S. (2019). Chlamydia, Gonorrhoea, Trichomoniasis and Syphilis: Global prevalence and incidence estimates, 2016. Bull. World Health Organ..

[3] de Vos L., Daniels J., Gebengu A., Mazzola L., Gleeson B., Piton J., Mdingi M., Gigi R., Ferreyra C., Klausner J.D. (2023). Usability of a novel Lateral Flow Assay for the Point-of-Care Detection of *Neisseria gonorrhoeae*: A Qualitative Time-Series Assessment among Healthcare Workers in South Africa. PLoS ONE.

[4] Ali A.S.M., Anderson C.S. (2024). Gepotidacin, a new first-in-class antibiotic for treating uncomplicated urinary tract infection. Lancet.

[5] Abdellati S., Laumen J.G.E., de Block T., De Baetselier I., Van Den Bossche D., Van Dijck C., Manoharan-Basil S.S., Kenyon C. (2024). Gonococcal resistance to zoliflodacin could emerge via transformation from commensal *Neisseria* species. An in-vitro transformation study. Sci. Rep..

[6] Fernandes P., Craft J.C. (2019). Phase 3 Trial of Treating Gonorrhoea with Solithromycin. Lancet Infect. Dis..

[7] Lyu M., Moseng M.A., Reimche J.L., Holley C.L., Dhulipala V., Su C.C., Shafer W.M., Yu E.W., Gilmore M.S. (2020). Cryo-EM Structures of a gonococcal multidrug efflux pump illuminate a mechanism of drug recognition and resistance. mBio.

[8] Jain N., Sk M.F., Mishra A., Kar P., Kumar A. (2022). Identification of novel efflux pump inhibitors for *Neisseria gonorrhoeae* via multiple ligand-based pharmacophores, e-pharmacophore, molecular docking, density functional theory, and molecular dynamics approaches. Comput. Biol. Chem..

[9] Hagman K.E., Lucas C.E., Balthazar J.T., Snyder L., Nilles M., Judd R.C., Shafer W.M. (1997). The MtrD protein of *Neisseria gonorrhaeae* is a member of the resistance/nodulation/division protein family constituting part of an efflux system. Microbiology.

[10] Li X.Z., Plésiat P., Nikaido H. (2015). The challenge of efflux-mediated antibiotic resistance in Gram-negative bacteria. Clin. Microbiol. Rev..

[11] Seukep A.J., Kuete V., Nahar L., Sarker S.D., Guo M. (2020). Plant-derived secondary metabolites as the main source of efflux pump inhibitors and methods for identification. J. Pharm. Anal..

[12] Adeosun I.J., Baloyi I.T., Cosa S. (2022). Anti-biofilm and associated anti-virulence activities of selected phytochemical compounds against *Klebsiella pneumoniae*. Plants.

[13] Heyman H.M., Senejoux F., Seibert I., Klimkait T., Maharaj V.J., Meyer J.J.M. (2015). Identification of anti-HIV active dicaffeoylquinic- and tricaffeoylquinic acids in *Helichrysum populifolium* by NMR-based metabolomic guided fractionation. Fitoterapia.

[14] DeRango-Adem E.F., Blay J. (2021). Does oral apigenin have real potential for a therapeutic effect in the context of human gastrointestinal and other cancers?. Front. Pharmacol..

[15] Jánosity A., Baranyi J., Surányi B.B., Možina S.S., Taczman-Brückner A., Kiskó G., Klančnik A. (2023). Estimating the optimal efflux inhibitor concentration of carvacrol as a function of the bacterial physiological state. Front. Microbiol..

[16] Adeosun I.J., Baloyi I., Aljoundi A.K., Salifu E.Y., Ibrahim M.A., Cosa S. (2022). Molecular modelling of SdiA protein by selected flavonoid and terpenes compounds to attenuate virulence in *Klebsiella pneumoniae*. J. Biomol. Struct. Dyn..

[17] Sadybekov A.V., Katritch V. (2023). Computational approaches streamlining drug discovery. Nature.

[18] Shimu M.S.S., Mahmud S., Tallei T.E., Sami S.A., Adam A.A., Acharjee U.K., Paul G.K., Emran T.B., Zaman S., Uddin M.S. (2022). Phytochemical compound screening to identify novel small molecules against Dengue virus: A docking and dynamics study. Molecules.

[19] Lamers R.P., Cavallari J.F., Burrows L.L. (2013). The efflux inhibitor phenylalanine-arginine beta-naphthylamide (PAβN) Permeabilizes the outer membrane of Gram-Negative bacteria. PLoS ONE.

[20] Pal A., Tripathi A. (2020). Quercetin inhibits carbapenemase and efflux pump activities among Carbapenem-resistant Gram-negative bacteria. Acta Pathol. Microbiol. Immunol. Scand..

[21] Sharma A., Gupta V.K., Pathania R. (2019). Efflux pump inhibitors for bacterial pathogens: From bench to bedside. Indian J. Med. Res..

[22] Alenazy R. (2022). Drug Efflux Pump Inhibitors: A promising approach to counter multidrug resistance in Gram-negative pathogens by targeting AcrB protein from AcrAB-TolC multidrug efflux pump from *Escherichia coli*. Biology.

[23] Gorlenko C.L., Kiselev H.Y., Budanova E.V., Zamyatnin A.A., Ikryannikova L.N. (2020). Plant secondary metabolites in the battle of drugs and drug-resistant bacteria: New heroes or worse clones of antibiotics?. Antibiotics.

[24] Gupta S., Bajaj A.V. (2018). Extra precision glide docking, free energy calculation, and molecular dynamics studies of 1,2-Diarylethane derivatives as potent urease inhibitors. J. Mol. Model..

[25] Celej M.S., Montich G.G., Fidelio G.D. (2003). Protein stability induced by Ligand Binding Correlates with Changes in Protein Flexibility. Protein Sci..

[26] Baloyi I.T., Adeosun I.J., Yusuf A.A., Cosa S. (2021). *In silico* and *in vitro* screening of antipathogenic properties of *Melianthus comosus* (Vahl) against *Pseudomonas aeruginosa*. Antibiotics.

[27] Kastritis P.L., Bonvin A.M.J.J. (2013). On the binding affinity of macromolecular interactions: Daring to ask why proteins interact. J. R. Soc. Interface.

[28] Meng X.-Y., Zhang H.-X., Mezei M., Cui M. (2012). Molecular docking: A powerful approach for structure-based drug discovery. Curr. Comput. Aided-Drug Des..

[29] Rácz A., Mihalovits L.M., Bajusz D., Héberger K., Miranda-Quintana R.A. (2022). Molecular dynamics simulations, and diversity selection by extended continuous similarity indices. J. Chem. Inf. Model..

[30] Lee K.S.S., Yang J., Niu J., Ng C.J., Wagner K.M., Dong H., Kodani S.D., Wan D., Morisseau C., Hammock B.D. (2019). Drug-target residence time affects *in vivo* target occupancy through multiple pathways. Am. Chem. Soc. Cent. Sci..

[31] Han J. (2022). Two-dimensional six-body van der Waals interactions. Atoms.

[32] Ultee A., Bennik M.H.J., Moezelaar R. (2002). The phenolic hydroxyl group of Carvacrol is essential for action against the food-borne pathogen *Bacillus cereus*. Appl. Environ. Microbiol..

[33] Childers M.C., Daggett V. (2017). Insights from molecular dynamics simulations for computational protein design. Mol. Syst. Des. Eng..

[34] Li Z., Chan K.C., Nickels J.D., Cheng X. (2022). Electrostatic contributions to the binding free energy of nicotine to the acetylcholine binding protein. J. Phys. Chem. B.

[35] Zhang S., Wang J., Ahn J. (2023). Advances in the discovery of efflux pump inhibitors as novel potentiators to control antimicrobial-resistant pathogens. Antibiotics.

[36] Adewumi A.T., Oluyemi W.M., Adekunle Y.A., Adewumi N., Alahmdi M.I., Soliman M.E.S., Abo-Dya N.E. (2023). Propitious indazole compounds as β-ketoacyl-ACP synthase inhibitors and mechanisms unfolded for TB cure: Integrated rational design and MD simulations. ChemistrySelect.

[37] Nguyen T.H.T., Nguyen H.D., Le M.H., Nguyen T.T.H., Nguyen T.D., Nguyen D.L., Nguyen Q.H., Nguyen T.K.O., Michalet S., Dijoux-Franca M.G. (2023). Efflux pump inhibitors in controlling antibiotic resistance: Outlook under a heavy metal contamination context. Molecules.

[38] Süzgeç-Selçuk S., Birteksöz A.S. (2011). Flavonoids of *Helichrysum chasmolycicum* and its antioxidant and antimicrobial activities. S. Afr. J. Bot..

[39] Bergström C.A.S., Larsson P. (2018). Computational prediction of drug solubility in water-based systems: Qualitative and quantitative approaches used in the current drug discovery and development setting. Int. J. Pharm..

[40] Tibbitts J., Canter D., Graff R., Smith A., Khawli L.A. (2016). Key factors influencing ADME properties of therapeutic proteins: A need for ADME characterization in drug discovery and development. mAbs.

[41] Mao F., Ni W., Xu X., Wang H., Wang J., Ji M., Li J. (2016). Chemical structure-related drug-like criteria of globally approved drugs. Molecules.

[42] Zotti M., Colaianna M., Morgese M.G., Tucci P., Schiavone S., Avato P., Trabace L. (2013). Carvacrol: From ancient flavoring to neuromodulatory agent. Molecules.

[43] de Andrés F., Altamirano-Tinoco C., Ramírez-Roa R., Montes-Mondragón C.F., Dorado P., Peñas-Lledó E.M., LLerena A. (2021). Relationships between CYP1A2, CYP2C9, CYP2C19, CYP2D6 and CYP3A4 metabolic phenotypes and genotypes in a Nicaraguan mestizo population. Pharmacogenomics J..

[44] Yang Z.Y., Yang Z.J., He J.H., Lu A.P., Liu S., Hou T.J., Cao D.S. (2021). Benchmarking the mechanisms of frequent hitters: Limitation of PAINS alerts. Drug Discov. Today.

[45] Pathania S., Singh P.K. (2021). Analyzing FDA-approved drugs for compliance of pharmacokinetic principles: Should there be a critical screening parameter in drug designing protocols?. Expert Opin. Drug Metab. Toxicol..

[46] Alves V.M., Muratov E.N., Capuzzi S.J., Politi R., Low Y., Braga R.C., Zakharov A.V., Sedykh A., Mokshyna E., Farag S. (2016). Alarms about structural alerts. Green Chem..

[47] Ben Arfa A., Combes S., Preziosi-Belloy L., Gontard N., Chalier P. (2006). Antimicrobial activity of carvacrol related to its chemical structure. Lett. Appl. Microbiol..

[48] Wijesundara N.M., Lee S.F., Cheng Z., Davidson R., Rupasinghe H.V. (2021). Carvacrol exhibits rapid bactericidal activity against *Streptococcus pyogenes* through cell membrane damage. Sci. Rep..

[49] Waditzer M., Bucar F. (2021). Flavonoids as inhibitors of bacterial efflux pumps. Molecules.

[50] Molina Bertrán S.D.C., Monzote L., Cappoen D., Escalona Arranz J.C., Gordillo Pérez M.J., Rodríguez-Ferreiro A.O., Chill Nuñez I., Novo C.P., Méndez D., Cos P. (2022). Inhibition of bacterial adhesion and biofilm formation by seed-derived ethanol extracts from *Persea americana* mill. Molecules.

[51] Martins M., McCusker M.P., Viveiros M., Couto I., Fanning S., Pagès J.M., Amaral L. (2013). A simple method for assessment of MDR bacteria for over-expressed efflux pumps. Open Microbiol. J..

[52] Sobhanipoor M.H., Ahmadrajabi R., Nave H.H., Saffari F. (2022). Determination of efflux activity in *Enterococci* by Hoechst accumulation assay and the role of zinc oxide nanoparticles in inhibition of this activity. BMC Microbiol..

[53] Pettersen E.F., Goddard T.D., Huang C.C., Couch G.S., Greenblatt D.M., Meng E.C., Ferrin T.E. (2004). UCSF Chimera—A visualization system for exploratory research and analysis. J. Comput. Chem..

[54] Genheden S., Ryde U. (2015). The MM/PBSA and MM/GBSA methods to estimate ligand-binding affinities. Expert Opin. Drug Discov..

[55] Deschenes L.A., David A. (2000). Vanden BoutUniversity of Texas, Austin Origin 6.0: Scientific data analysis and graphing software origin lab corporation (formerly microcal software, Inc.). Web Site: Www.Originlab.Com. Commercial Price: $595. Academic Price: $446. J. Am. Chem. Soc..

[56] Kowalska-Krochmal B., Dudek-Wicher R. (2021). The minimum inhibitory concentration of antibiotics: Methods, interpretation, clinical relevance. Pathogens.

[57] Zhang G.F., Liu X., Zhang S., Pan B., Liu M.L. (2018). Ciprofloxacin derivatives and their antibacterial activities. Eur. J. Med. Chem..

